# Hesitant 2-tuple fuzzy linguistic multi-criteria decision-making method based on correlation measures

**DOI:** 10.1371/journal.pone.0270414

**Published:** 2022-08-09

**Authors:** Muhammad Sajjad, Wojciech Sałabun, Shahzad Faizi, Muhammad Ismail

**Affiliations:** 1 Department of Statistics, Comsats University Islamabad, Lahore, Pakistan; 2 Research Team on Intelligent Decision Support Systems, Department of Artificial Intelligence Methods and Applied Mathematics, Faculty of Computer Science and Information Technology, West Pomeranian University of Technology in Szczecin, Szczecin, Poland; 3 Department of Mathematics, Virtual University of Pakistan, Lahore, Pakistan; PLOS (Public Library of Science), UNITED KINGDOM

## Abstract

Correlation is considered the most important factor in analyzing the data in statistics. It is used to measure the movement of two different variables linearly. The concept of correlation is well-known and used in different fields to measure the association between two variables. The hesitant 2-tuple fuzzy linguistic set (H2FLS) comes out to be valuable in addressing people’s reluctant subjective data. The purpose of this paper is to analyze new correlation measures between H2FLSs and apply them in the decision-making process. First and foremost, the ideas of mean and variance of hesitant 2-tuple fuzzy linguistic elements (H2FLEs) are introduced. Then, a new correlation coefficient between H2FLSs is established. In addition, considering that different H2FLEs may have different criteria weights, the weighted correlation coefficient and ordered weighted correlation coefficient are further investigated. A practical example concerning the detailed procedure of solving problems is exemplified to feature the reasonableness and attainability of the proposed technique in situations where the criteria weights are either known or unknown. When the weight vector is unknown, the best-worst method (BWM) is used to acquire the criteria weights in the context of a hesitant 2-tuple fuzzy linguistic environment. Furthermore, a comparative study is undertaken with current techniques to provide a vision into the design decision-making process. Finally, it is verified that the proposed correlation coefficient between H2FLSs is more satisfactory than the extant ones, and the correlation coefficient with the weights of criteria being either known or unknown is applicable.

## 1 Introduction

We mostly interact with multi-criteria group decision-making (MCGDM) problems in our routine life, which are used to choose the best alternatives among different alternatives. Often we deal with dubious situations, and due to the ambiguous nature of MCGDM problems, the fuzzy method may be considered as a valid technique to define the characteristics like linguistic variables [1–3], interval values [4, 5] and hesitant fuzzy numbers [6]. We have to deal with uncertainty at different levels. The two most important approaches introduced to investigate, interrogate, and tackle this type of uncertainty are fuzzy set theory and probability theory. For the first time, Zadeh [7] characterized the fuzzy linguistic techniques to manage the decision-making problems, which utilizes linguistic factors whose contents are not quantities, but phrases or terms, to address subjective assessments. After the development of the linguistic approach, many researchers took an interest in this area and applied it to MCGDM problems [8]. Over time, researchers feel some limitation in the linguistic variable: it can only express the membership degree. There are many situations where consideration of membership and non-membership functions is necessary.

For some dynamic issues, it is simple for decision-makers or experts to communicate their assessments in phonetic or linguistic terms, for example, “low” cost, “high” quality, “great” execution. The linguistic variable upgrades the possibility and adaptability of decision models, and the advancement of analyzing linguistic variables have prompted a new research territory named computing with words [7]. However, the customary linguistic methodology uses a one-word linguistic term to evaluate a linguistic variable. This way cannot represent the hesitant impression of decision-makers. For example, while assessing a research proposal, a decision-maker may state, “it is between good and excellent”. Also, in a group decision-making process, the gathering’s appraisals could be uncertain if there are various assessments among many individuals. To overthrow the inefficiency of the linguistic process, Rodríguez et al. [9] introduced the concept of hesitant fuzzy linguistic term set (HFLTS), where decision-makers can give their ambiguous evaluations using several linguistic terms. Consequently, the HFLTS has pulled in numerous scholars’ attention [10, 11].

Herrera and Martins [12, 13] proposed a 2-tuple linguistic representation model that can deal with verbal and numerical data viably in decision-making without mutilating and harming data. This helpful model is basically founded on the symbolic translation of linguistic variables and has been generally utilized in different MCDM problems [14]. In light of the difficulty of items and dubiousness of individuals thinking, it is increasingly suitable to give an evaluation by using linguistic data rather than numerical numbers [15, 16]. Accordingly, the number of analysts dealt with linguistic data for taking care of MCGDM issues and characterized various techniques to explore the linguistic assessment components. There are mainly three parts of the linguistic information model [16, 17], procedure based on membership degree, procedure based on linguistic symbols, and the procedure based in linguistic 2-tuple. However, the initial two techniques have a few restrictions that they played out their interpretation procedure as an estimation procedure to assess the finding in the original space, thus invigorating the absence of exactness and precision [18]. Contrarily, the precision of linguistic terms, calculation, and interpretation of results can be improved with the assistance of the 2-tuple linguistic model. In light of its recognized quality and viability in managing the linguistic judgment of experts, the 2-tuple linguistic models have been generally utilized in decision-making problems [19–21]. Zhang et al. [22] examined the transitivity of 2-tuple to tackle the issue of group decision-making problems. Apart from this, the idea of a hesitant 2-tuple fuzzy linguistic set is proposed by Wei and Liao [23]. Beg and Rashid [14] extended fuzzy TOPSIS for hesitant 2-tuple linguistic term sets with the assessment of some experts about the properties of alternatives. Wang et al. [24] built up a hesitant 2-tuple linguistic aggregation operator for structuring a new type of group decision-making approach to deal with addressing the data combination, including the interrelationship between aggregated terms and the prioritization relationship among experts under hesitant 2-tuple linguistic circumstance.

At whatever point we talk about the strength of the relationship between two variables, the correlation coefficient is a suitable measurement tool. In all estimation procedures, the correlation coefficient is an effective apparatus and broadly utilized in statistical analysis to quantify the strength or level of relationship between two variables [25–27]. However, it is conspicuous that the idea constructed on daily life problems is uncertain. So as to handle this issue, correlation is expanded by different analysts to fuzzy correlation [28–30]. Tyagi [31] broadened the idea of the hesitant fuzzy set to dual hesitant fuzzy set and built up a correlation coefficient between dual hesitant fuzzy sets. They applied it to assess the correlation coefficient estimation among various water parameters of four distinct lakes. A correlation coefficient based on the central interval is proposed by Saneifard [27]. Chen et al. [32] outlined a correlation coefficient for hesitant fuzzy sets and applied it to clustering analysis in a hesitant fuzzy environment. Moreover, they likewise proposed some interval-valued hesitant fuzzy sets, introduced their correlation coefficients, and determined their applications in clustering. Arora and Garg [33] took inspiration from the soft set theory and built up the correlation coefficient to measure the relationship between dual hesitant fuzzy soft sets, named the novel correlation coefficient.

MCDM is a developing field of study that helps experts distinguish the best alternative from a set of alternatives assessed through a set of criteria weights [34]. A basic portion of this issue is the arrangement of the significance of criteria weights. Various methods have been developed to recognize these criteria, including [35–37], SMARTS and SMARTER, analytic hierarchy process. BWM is also linked to them, designed by Rezaie [38] that depends on an organized pairwise comparison framework for an optimal model to get the ideal weights of criteria. This method has fascinated scholars’ attention from different fields since its presentation. Hadi et al. [39] presented z-number BWM to deal with the uncertain situation of MCGDM problems. Mi et al. [40] presented some questions relating to BWM in the form of why what, what for, and what next. Muhammad & Razaei [41] introduced a Bayesian BWM to find the aggregated final criteria weights of models for a group of decision-makers. Furthermore, many other works are available in the literature on BWM, including multiplicative BWM [42], BWM MULTIMOORA [43], BWM VIKOR [44], and others [45].

Adopting inspiration from the existing approaches [24, 46], a new type of correlation coefficient between H2FLSs is proposed in this paper. Numerous methodologies exist in the literature that works in information energy and develops a recipe for computing the correlation coefficient. However, there was no technique for estimating the distance-based correlation coefficient from a statistics point of view in the context of H2FLSs. The proposed correlation coefficient can determine how strong the relationship between the two alternatives/variables is. It reveals information about the direction of the relationship and can also display data behaviour that is difficult to study by simple observation. However, drawbacks are also related, such as the inability to determine the curvilinear relationship. Furthermore, it is unclear which alternatives/variables are most affected in the correlation process and which ones are dependent and independent alternatives/variables. It also has no control over the third alternatives/variables influencing the relationship.

### 1.1 Research challenges and gaps

Correlation coefficients are utilized to show, in a linear design, the closeness level of a couple of variables. The correlation measures can be characterized into two classes, i.e., the statistics based correlation whose findings have a place in the interval [−1, 1] [46], and the information energy-based correlation measures, whose results vary inside the interval [0, 1] [26, 30]. Many researchers developed different types of correlation coefficients, as discussed in the introduction, but we still need a statistics-based correlation coefficient that deals H2FLSs. For example, for hesitant fuzzy linguistic term sets, [26, 46] suggested correlation coefficients, while [30] proposed a correlation coefficient for dual hesitant fuzzy linguistic term sets. Aside from these methods, there are other alternative correlation coefficients for dealing with various extensions of fuzzy sets, but none was for dealing with H2FLSs. Therefore, there was a challenging work that no technique for estimating the correlation coefficient from a statistics standpoint in the context of H2FLSs is available.

### 1.2 Contributions of this study

In this study, we explore the correlation measure between H2FLSs from a statistical perspective. The theoretical commitments of this paper can be summed up as follows:

- We proposed the idea of the mean and variance of the H2FLEs.
- We propose a new correlation coefficient between H2FLSs, whose findings lie in the interval [−1, 1].

The proposed correlation coefficient between H2FLSs can be utilized to show the positive or negative correlation between two variables in the context of a hesitant 2-tuple fuzzy linguistic environment. It overcomes the limitation of the previous correlation coefficients proposed in [26, 30], accordingly is significantly more persuading and has more extensive application possibilities. Practically, the proposed correlation coefficient can be applied in many real-life group decision-making problems, like engineering, medical studies, business studies, etc. The proposed approach is simple to implement, but there are some limitations. For example, the calculations may take longer to complete if the number of alternatives is large during the computation. Furthermore, unlike the conventional correlation coefficient, the proposed correlation coefficient is not independent of origin and scale. It also does not indicate the cause and effect relationship between the alternatives.

Furthermore, the evaluation of H2FLEs for which the weights of criteria are either totally known or unknown is discussed. The BWM approach is used to evaluate the criteria weights for the second circumstance. They are altogether more convincing than the abstract weight-deciding methodology that experts straightforwardly give the weights.

### 1.3 Framework of this study

The overall sequence of this paper is prescribed as follows: some preliminary definitions and existing correlation coefficients are briefly discussed in section 2. The proposed method for H2FLSs with some important properties is discussed in section 3. A brief discussion of BWM is mentioned in section 4. The validity of the work illustrated with the help of example is given in section 5. Comparative analysis with some existing approaches is described in section 6. At long last, the consequence is given at the end.

## 2 Preliminaries

In this section, we discuss some basic notions of linguistic term set (LTS), hesitant fuzzy LTS, 2-tuple linguistic model and H2FLS.

**Definition 1** [47, 48] Suppose, the LTS *S* = {*u*_0_, *u*_1_, …, *u*_*g*_}, has an odd cardinality, where *g* is even and *u*_*i*_ ∈ [0, *g*]. The set *S* has the given two characteristics:

1. Negation operator: *Neg*(*u*_*i*_) = *u*_*j*_, such that *i* + *j* = *g*;
2. Ordered set: *u*_*i*_ ≤ *u*_*j*_ ⇔ *i* ≤ *j*. thus, there exist a maximum and minimum operators i.e.
  - max(*u*_*i*_, *u*_*j*_) = *u*_*i*_, if *u*_*j*_ ≤ *u*_*i*_;
  - min(*u*_*i*_, *u*_*j*_) = *u*_*i*_, if *u*_*i*_ ≤ *u*_*j*_.

The continuous LTS $\bar{S}$ is defined by Xu [49, 50], which is an extension of the discrete term set *S* where $\bar{S}=\left\{u_{k}|u_{0}\leq u_{k}\leq u_{g}\right\}$. If *u*_*k*_ ∈ *S*, *u*_*k*_ is called the original linguistic term and will be used to assess the alternatives throughout a decision process. If *u*_*k*_ ∉ *S*, then *u*_*k*_ is called the virtual linguistic term of *S* that will appear only in the calculations process.

**Definition 2** [9] Let *x*_*i*_ ∈ *X*, *i* = 1, 2, ⋯, *N*, be fixed and *S* = {*s*_*t*_|*t* = −*τ*, ⋯, −1, 0, 1, ⋯, *τ*} be a LTS. A hesitant fuzzy LTS on *X*, *H*_*S*_, is in the form of

$$\begin{array}{c} H_{S}=\{<x_{i},h_{S}\left(x_{i}\right)>|xi\in X\}, \end{array}$$

where $h_{S}\left(x_{i}\right)=\{s_{\varphi_{l}}\left(xi\right)|s_{\varphi_{l}}\left(xi\right)\in S;l=1,2,...,L\left(x_{i}\right)\}$ with *L*(*x*_*i*_) being the number of LTS in *h*_*S*_(*x*_*i*_) and *φ*_*l*_ ∈ [−*τ*, *τ*].

Herrera and Martínez [12] defined 2-tuple linguistic model to represent the linguistic type of data by 2-tuple (*u*_*i*_, *a*), where *u*_*i*_ ∈ *S* and *a* ∈ [−0.5, 0.5). It means that, a function is transformed into 2-tuple linguistic term and a numerical number.

**Definition 3** [12] Let $S=\{u_{0},u_{1},...,\;u_{g}\}$ be a LTS and *β* ∈ [0, *g*] is a numerical value indicating the result of a symbolic aggregation operator. Then, △ : [0, *g*] → *S*× [-0.5, 0.5) shows a 2-tuple linguistic function that presenting the same information to *β* is defined as follows:

$$\begin{array}{c} △\left(\beta \right)=\left(u_{i},\alpha \right),\text{with}\{\begin{array}{cc} u_{i},\;\;\; & i=\text{round}\;(\beta ),\\ \alpha =\beta -i, & \alpha \in [-0.5,0.5). \end{array} \end{array}$$

△ operator is a one-to-one function and an inverse function is ▽((*u*_*i*_, *a*)) = *i* + *a*.

The 2-tuple linguistic model ought to be intelligible and predefined, by which each decision-maker can additionally qualify potential alternatives for specific criteria. However, because of the complication of objects and vagueness of people thinking, the 2-tuple linguistic model can not wholly describe the hesitating circumstances. Therefore, Wei and Liao [23] presented the hesitant 2-tuple fuzzy linguistic data model to deal with the situations in which data portrayed is in linguistic terms, and the decision-maker has some hesitation in choosing its conceivable linguistic interpretations. It can deal with the information viably in decision-making without harming data and more accurate results. Wei and Liao [23] defined the H2FLS as follows.

**Definition 4** [23] Let *S* = {*u*_0_, *u*_1_, …, *u*_*g*_} be a LTS. A H2FLS on *X*, *H*_*S*_, is in the form of *H*_*S*_ = {< *x*_*i*_, *h*(*x*_*i*_) > ; *x*_*i*_ ∈ *X*)}, where *h*(*x*_*i*_) = {*β*_*i*_ = (*u*_*i*_, *α*_*i*_)|*i* = 1, 2, …, #*h*(*x*_*i*_)} and *α* ∈ [−0.5, 0.5). For convenience *h*(*x*_*i*_) is called a H2FLE.

In our next study, this hesitant 2-tuple fuzzy linguistic data model will be used in our research for the construction of correlation coefficient for H2FLEs.

## 3 Mean and variance of H2FLEs

In this section, we established the mean and variance for H2FLEs. The developed mean and variance will further use in the construction of the correlation coefficient for H2FLSs. It can be defined as follows:

**Definition 5** Let *S* = {*u*_0_, *u*_1_, …, *u*_*g*_} be a LTS and *h*(*x*) = *β*_*i*_, be a H2FLEs. The mean of *h*(*x*) is defined as:

$$\begin{array}{c} \bar{h}=\frac{1}{\#h}\sum_{i=1}^{\#h}▽\beta_{i} \end{array}$$ (1)

where *β*_*i*_ = (*u*_*i*_, *α*_*i*_) and *i* = 1, 2, …, #*h*. The variance of *h*(*x*) is defined as:

$$\begin{array}{c} Var\left(h\right)=\frac{1}{\#h}\sum_{i=1}^{\#h}{\left(▽\beta_{i}-\bar{h}\right)}^{2} \end{array}$$ (2)

**Example:** For a LTS *S* = {*u*_0_, *u*_1_, …, *u*_6_}, suppose *h*^1^ = ((*u*_3_, 0.2487), (*u*_1_, 0.2752)), *h*^2^ = ((*u*_2_, 0.0477), (*u*_1_, 0.1427), (*u*_1_, 0.2752)) and *h*^3^ = ((*u*_4_, 0.2487), (*u*_1_, 0.2251)) are three H2FLEs. By using Eqs 1 and 2, the mean and variance of *h*^1^, *h*^2^ and *h*^3^ are computed as ${\bar{h}}^{1}=2.2619,$ ${\bar{h}}^{2}=1.4885$, ${\bar{h}}^{3}=2.7369$, *Var*(*h*^1^) = 0.9734, *Var*(*h*^2^) = 0.1592 and *Var*(*h*^3^) = 2.2855.

### 3.1 Covariance and correlation coefficient of H2FLSs

In the present subsection, covariance and correlation coefficient are discussed. The covariance of two random variables is a numerical measure of the extent to which their values tend to increase or decrease together. As the estimations of covariance depend on the scale of measurement for *x* and *y*, and afterwards, it must be standardized before it’s utilization like a conventionally practical measure of association, so it is not a reasonable descriptive measure to compute the relationship between two variables, that is the reason, the correlation coefficient is used to measure the relationship between two variables. The measure of correlation is used in many situations for decision-making problems. Therefore, this paper is based on statistics, which measures the correlation coefficient for H2FLSs.

**Definition 6** Let *S* = {*u*_0_, *u*_1_, …, *u*_*g*_} be a LTS. A H2FLS on *X*, *H*_*S*_, is in the form of *H*_*S*_ = {< *x*_*i*_, *h*^*i*^(*x*_*i*_) > ; *x*_*i*_ ∈ *X*)}, where *h*^*i*^(*x*_*i*_) = {(*β*_*i*_ = (*u*_*i*_(*x*_*i*_), *α*(*x*_*i*_))|*i* = 1, 2, …, #*h*(*x*_*i*_)} and *α* ∈ [−0.5, 0.5). The mean of H2FLS can be defined as:

$$\begin{array}{c} {\bar{H}}_{S}=\frac{1}{n}\sum_{i=1}^{n}{\bar{h}}^{i} \end{array}$$ (3)

where ${\bar{h}}^{i}=\frac{1}{\#h^{i}}\sum_{i=1}^{\#h^{i}}▽\beta_{i}$. The variance of the H2FLS is defined as:

$$\begin{array}{c} Var\left(H_{S}\right)=\frac{1}{n}\sum_{i=1}^{n}{\left({\bar{h}}^{i}-{\bar{H}}_{S}\right)}^{2} \end{array}$$ (4)

**Definition 7** Let *S* = {*u*_0_, *u*_1_, …, *u*_*g*_} be a LTS. For two H2FLSs $H_{S}^{a}=\left\{<x_{i},h^{ai}\left(x_{i}\right)>;x_{i}\in X\right)\}$ and $H_{S}^{b}=\left\{<x_{i},h^{bi}\left(x_{i}\right)>;x_{i}\in X\right)\}$ where $h^{ai}\left(x_{i}\right)=(\beta_{i}^{a}=\left(u_{i}^{a}\left(x_{i}\right),\alpha^{a}\left(x_{i}\right)\right)|i=1,2,...,\#h^{ai}\left(x_{i}\right))$, $h^{bi}\left(x_{i}\right)=\beta_{i}^{b}=\left(\left(u_{i}^{b}\left(x_{i}\right),\alpha^{b}\left(x_{i}\right)\right)|i=1,2,...,\#h^{bi}\left(x_{i}\right)\right)$ and *α*(*x*_*i*_) ∈ [−0.5, 0.5). The covariance between $H_{S}^{a}$ and $H_{S}^{b}$ is defined as:

$$\begin{array}{c} C(H_{S}^{a},H_{S}^{b})=\frac{1}{n}\sum_{i=1}^{n}({\bar{h}}^{ai}-{\bar{H}}_{S}^{a})({\bar{h}}^{bi}-{\bar{H}}_{S}^{b}) \end{array}$$ (5)

**Definition 8** For two H2FLSs, $H_{S}^{a}$ and $H_{S}^{b}$ as defined before. The correlation coefficient between $H_{S}^{a}$ and $H_{S}^{b}$ is defined as:

$$\begin{array}{c} \rho \left(H_{S}^{a},H_{S}^{b}\right)=\frac{\sum_{i=1}^{n}({\bar{h}}^{ai}-{\bar{H}}_{S}^{a})({\bar{h}}^{bi}-{\bar{H}}_{S}^{b})}{\sqrt{\sum_{i=1}^{n}{\left({\bar{h}}^{ai}-{\bar{H}}_{S}^{a}\right)}^{2}}\sqrt{\sum_{i=1}^{n}{\left({\bar{h}}^{bi}-{\bar{H}}_{S}^{b}\right)}^{2}}} \end{array}$$ (6)

### 3.2 Important properties

(i) $C(H_{S}^{a},H_{S}^{a})=Var(H_{S}^{a})$
(ii) $C(H_{S}^{a},H_{S}^{b})=C(H_{S}^{b},H_{S}^{a})$
(iii) $C{\left(H_{S}^{a},H_{S}^{b}\right)}^{2}\leq \sqrt{Var(H_{S}^{a})}\sqrt{Var(H_{S}^{b})}$
(iv) $\rho \left(H_{S}^{a},H_{S}^{b}\right)=\rho \left(H_{S}^{b},H_{S}^{a}\right)$
(v) $\rho (H_{S}^{a},H_{S}^{a})=1$
(vi) $-1\leq \rho (H_{S}^{a},H_{S}^{b})\leq 1$
(vii) If $H_{S}^{a}=cH_{S}^{b},$ then $\rho \left(H_{S}^{a},H_{S}^{b}\right)=\{\begin{array}{cc} 1; & c>0\\ -1; & c<0 \end{array}$

**Proof (i)** $C(H_{S}^{a},H_{S}^{a})$ $=Var(H_{S}^{a})$
  $$\begin{array}{c} C(H_{S}^{a},H_{S}^{a})=\frac{1}{n}\sum_{i=1}^{n}({\bar{h}}^{ai}-{\bar{H}}_{S}^{a})({\bar{h}}^{ai}-{\bar{H}}_{S}^{a}) \end{array}$$
  $$\begin{array}{c} C(H_{S}^{a},H_{S}^{a})=\frac{1}{n}\sum_{i=1}^{n}{\left({\bar{h}}^{ai}-{\bar{H}}_{S}^{a}\right)}^{2}=Var\left(H_{S}^{a}\right) \end{array}$$
**Proof (ii)** is obvious, so we omit here the proof.
**Proof (iii)** $C{\left(H_{S}^{a},H_{S}^{b}\right)}^{2}\leq \sqrt{Var(H_{S}^{a})}\sqrt{Var(H_{S}^{b})}$
  $$\begin{array}{c} C(H_{S}^{a},H_{S}^{b})=\frac{1}{n}\sum_{i=1}^{n}([{\bar{h}}^{ai}-{\bar{H}}_{S}^{a}])({\bar{h}}^{bi}-{\bar{H}}_{S}^{b}) \end{array}$$
  $$\begin{array}{c} C(H_{S}^{a},H_{S}^{b})=[\left({\bar{h}}^{a1}-{\bar{H}}_{S}^{a}\right)...\left({\bar{h}}^{an}-{\bar{H}}_{S}^{a}\right)][\left({\bar{h}}^{b1}-{\bar{H}}_{S}^{b}\right)...\left({\bar{h}}^{bn}-{\bar{H}}_{S}^{b}\right)] \end{array}$$
  $$\begin{array}{c} C(H_{s}^{a},H_{s}^{b})={\left|\begin{array}{c} {}[(\left({\bar{h}}^{a1}-{\bar{H}}_{S}^{a}\right)\left({\bar{h}}^{a1}-{\bar{H}}_{S}^{a}\right)+...+\left({\bar{h}}^{an}-{\bar{H}}_{S}^{a}\right)\left({\bar{h}}^{an}-{\bar{H}}_{S}^{a}\right)]*\\ \left[\left({\bar{h}}^{b1}-{\bar{H}}_{S}^{b}\right)\left({\bar{h}}^{b1}-{\bar{H}}_{S}^{b}\right)+...+\left({\bar{h}}^{bn}-{\bar{H}}_{S}^{b}\right)\left({\bar{h}}^{bn}-{\bar{H}}_{S}^{b}\right)\right] \end{array}\right|}^{1/2} \end{array}$$
  According to Cauchy-Schwartz inequality
  $$\begin{array}{c} C{\left(H_{S}^{a},H_{S}^{b}\right)}^{2}\leq {\left|[{\left({\bar{h}}^{a1}-{\bar{H}}_{S}^{a}\right)}^{2}+,...,+{\left({\bar{h}}^{an}-{\bar{H}}_{S}^{a}\right)}^{2}][{\left({\bar{h}}^{b1}-{\bar{H}}_{S}^{b}\right)}^{2}+,...,+{\left({\bar{h}}^{bn}-{\bar{H}}_{S}^{b}\right)}^{2}]\right|}^{1/2} \end{array}$$
  $$\begin{array}{c} C{\left(H_{S}^{a},H_{S}^{b}\right)}^{2}\leq \sqrt{\frac{1}{n}\sum_{i=1}^{n}{\left({\bar{h}}^{ai}-{\bar{H}}_{S}^{a}\right)}^{2}}\sqrt{\frac{1}{n}\sum_{i=1}^{n}{\left({\bar{h}}^{bi}-{\bar{H}}_{S}^{b}\right)}^{2}}=\sqrt{Var(H_{S}^{a})}\sqrt{Var(H_{S}^{b})} \end{array}$$
**Proof (iv)** is obvious, so we omit here the proof.
**Proof (v)** $\rho (H_{S}^{a},H_{S}^{a})=1$
  $$\begin{array}{c} \rho \left(H_{S}^{a},H_{S}^{a}\right)=\frac{\sum_{i=1}^{n}({\bar{h}}^{ai}-{\bar{H}}_{S}^{a})({\bar{h}}^{ai}-{\bar{H}}_{S}^{a})}{\sqrt{\sum_{i=1}^{n}{\left({\bar{h}}^{ai}-{\bar{H}}_{S}^{a}\right)}^{2}}\sqrt{\sum_{i=1}^{n}{\left({\bar{h}}^{ai}-{\bar{H}}_{S}^{a}\right)}^{2}}} \end{array}$$
  $$\begin{array}{c} \rho \left(H_{S}^{a},H_{S}^{a}\right)=\frac{\sum_{i=1}^{n}{\left({\bar{h}}^{ai}-{\bar{H}}_{S}^{a}\right)}^{2}}{\sum_{i=1}^{n}{\left({\bar{h}}^{ai}-{\bar{H}}_{S}^{a}\right)}^{2}}=1 \end{array}$$
**Proof (vi)** $-1\leq \rho (H_{S}^{a},H_{S}^{b})\leq 1$
  The most important property of the correlation coefficient lies between -1 and 1, inclusive. We will prove this property for the special case where we have random variables with zero means and unit variances.
  To prove this property, we standardize the two variables as
  $$H_{S}^{a}=\frac{\left({\bar{h}}^{ai}-\;{\bar{H}}_{S}^{a}\right)}{\sqrt{\sum_{i=1}^{n}{\left({\bar{h}}^{ai}-\;{\bar{H}}_{S}^{a}\right)}^{2}}}\;\text{and}\;H_{S}^{b}=\frac{\left({\bar{h}}^{bi}-\;{\bar{H}}_{S}^{b}\right)}{\sqrt{\sum_{i=1}^{n}{\left({\bar{h}}^{bi}-\;{\bar{H}}_{S}^{b}\right)}^{2}}}.$$
  It is obvious that the expected values or means and variances of the two variables are 0 and 1, respectively. Symbolically, we write them mathematically as:
  $$E(H_{S}^{a})=E(H_{S}^{b})=0\;\text{and}\;Var\left(H_{S}^{a}\right)=Var\left(H_{S}^{b}\right)=1.$$
  We also know that
  $$Var\left(H_{S}^{a}\pm H_{S}^{b}\right)=Var\left(H_{S}^{a}\right)+Var\left(H_{S}^{b}\right)\pm 2C\left(H_{S}^{a},H_{S}^{b}\right)$$
  But $C\left(H_{S}^{a},H_{S}^{b}\right)=E\left(H_{S}^{a}H_{S}^{b}\right)-E\left(H_{S}^{a}\right)E\left(H_{S}^{b}\right)=E\left(H_{S}^{a}H_{S}^{b}\right)$ $\left(∵E\left(H_{S}^{a}\right)=E\left(H_{S}^{b}\right)=0\right)$
  $$\Rightarrow E[\frac{\left(h^{a}-E\left(H_{S}^{a}\right))(h^{b}-E\left(H_{S}^{b}\right)\right)}{\sqrt{Var\left(H_{S}^{b}\right)Var\left(H_{S}^{b}\right)}}]=\rho =\frac{\sum_{i=1}^{n}({\bar{h}}^{ai}-{\bar{H}}_{S}^{a})({\bar{h}}^{bi}-{\bar{H}}_{S}^{b})}{\sqrt{\sum_{i=1}^{n}{\left({\bar{h}}^{ai}-{\bar{H}}_{S}^{a}\right)}^{2}}\sqrt{\sum_{i=1}^{n}{\left({\bar{h}}^{bi}-{\bar{H}}_{S}^{b}\right)}^{2}}}$$
  Thus
  $$Var\left(H_{S}^{a}+H_{S}^{b}\right)=1+1+2\rho =1+1+2[\frac{\sum_{i=1}^{n}({\bar{h}}^{ai}-{\bar{H}}_{S}^{a})({\bar{h}}^{bi}-{\bar{H}}_{S}^{b})}{\sqrt{\sum_{i=1}^{n}{\left({\bar{h}}^{ai}-{\bar{H}}_{S}^{a}\right)}^{2}}\sqrt{\sum_{i=1}^{n}{\left({\bar{h}}^{bi}-{\bar{H}}_{S}^{b}\right)}^{2}}}]$$
  $$=2\left(1+\rho \right)=2(1+\frac{\sum_{i=1}^{n}({\bar{h}}^{ai}-{\bar{H}}_{S}^{a})({\bar{h}}^{bi}-{\bar{H}}_{S}^{b})}{\sqrt{\sum_{i=1}^{n}{\left({\bar{h}}^{ai}-{\bar{H}}_{S}^{a}\right)}^{2}}\sqrt{\sum_{i=1}^{n}{\left({\bar{h}}^{bi}-{\bar{H}}_{S}^{b}\right)}^{2}}})\geq 0$$
  since $Var(H_{S}^{a}+H_{S}^{b})$ must be non-negative.
  $$\Rightarrow \frac{\sum_{i=1}^{n}({\bar{h}}^{ai}-{\bar{H}}_{S}^{a})({\bar{h}}^{bi}-{\bar{H}}_{S}^{b})}{\sqrt{\sum_{i=1}^{n}{\left({\bar{h}}^{ai}-{\bar{H}}_{S}^{a}\right)}^{2}}\sqrt{\sum_{i=1}^{n}{\left({\bar{h}}^{bi}-{\bar{H}}_{S}^{b}\right)}^{2}}}\geq -1$$
  Similarly $Var\left(H_{S}^{a}-H_{S}^{b}\right)=2\left(1-\rho \right)$
  $$=2(1-\frac{\sum_{i=1}^{n}({\bar{h}}^{ai}-{\bar{H}}_{S}^{a})({\bar{h}}^{bi}-{\bar{H}}_{S}^{b})}{\sqrt{\sum_{i=1}^{n}{\left({\bar{h}}^{ai}-{\bar{H}}_{S}^{a}\right)}^{2}}\sqrt{\sum_{i=1}^{n}{\left({\bar{h}}^{bi}-{\bar{H}}_{S}^{b}\right)}^{2}}})$$
  $$\Rightarrow \frac{\sum_{i=1}^{n}({\bar{h}}^{ai}-{\bar{H}}_{S}^{a})({\bar{h}}^{bi}-{\bar{H}}_{S}^{b})}{\sqrt{\sum_{i=1}^{n}{\left({\bar{h}}^{ai}-{\bar{H}}_{S}^{a}\right)}^{2}}\sqrt{\sum_{i=1}^{n}{\left({\bar{h}}^{bi}-{\bar{H}}_{S}^{b}\right)}^{2}}}\leq 1.$$
  Hence from these two results, we get
  $$-1\leq \frac{\sum_{i=1}^{n}({\bar{h}}^{ai}-{\bar{H}}_{S}^{a})({\bar{h}}^{bi}-{\bar{H}}_{S}^{b})}{\sqrt{\sum_{i=1}^{n}{\left({\bar{h}}^{ai}-{\bar{H}}_{S}^{a}\right)}^{2}}\sqrt{\sum_{i=1}^{n}{\left({\bar{h}}^{bi}-{\bar{H}}_{S}^{b}\right)}^{2}}}\leq 1$$
  $$\Rightarrow -1\leq \rho (H_{S}^{a},H_{S}^{b})\leq 1.$$
**Proof (vii)** Let $H_{S}^{a}=cH_{S}^{b},$ then for *c* > 0
  $$\frac{\sum_{i=1}^{n}(c{\bar{h}}^{bi}-c{\bar{H}}_{S}^{b})({\bar{h}}^{bi}-{\bar{H}}_{S}^{b})}{\sqrt{\sum_{i=1}^{n}{\left(c{\bar{h}}^{bi}-c{\bar{H}}_{S}^{b}\right)}^{2}}\sqrt{\sum_{i=1}^{n}{\left({\bar{h}}^{bi}-{\bar{H}}_{S}^{b}\right)}^{2}}}=\frac{c\sum_{i=1}^{n}{\left({\bar{h}}^{bi}-{\bar{H}}_{S}^{b}\right)}^{2}}{\sqrt{c^{2}\sum_{i=1}^{n}{\left({\bar{h}}^{bi}-{\bar{H}}_{S}^{b}\right)}^{2}}}=1$$
  $$\Rightarrow \rho (H_{S}^{a},H_{S}^{b})=1$$
  Similarly, for *c* < 0, $\rho (H_{S}^{a},H_{S}^{b})=-1$
  Hence $\rho \left(H_{S}^{a},H_{S}^{b}\right)=\{\begin{array}{cc} 1;\;\;\; & c>0\\ -1; & c<0 \end{array}$

### 3.3 Weighted correlation measures of H2FLSs

It has been observed that arguments about different objects occasionally can be linked with different weights. Hence, while computing the correlation coefficient between H2FLSs, we will consider the influence found from the weights. In the current section, we developed a weighted correlation coefficient in the hesitant 2-tuple fuzzy linguistic environment.

Suppose that the weight associated with each objective *x*_*i*_ is *ω*_*i*_, where *ω*_*i*_ ∈ [0, 1] (*i* = 1, 2, …, *n*) and $\sum_{i=1}^{n}\omega_{i}=1.$

1. The weighted mean of the H2FLS is:
  $${\bar{H}}_{S(\omega )}=\frac{1}{n}\sum_{i=1}^{n}(\omega_{i}{\bar{h}}^{i})$$
2. The weighted variance of the H2FLS is:
  $$Var_{\omega }\left(H_{S}\right)=\frac{1}{n}\sum_{i=1}^{n}{\left(\omega_{i}{\bar{h}}^{i}-{\bar{H}}_{S}\right)}^{2}$$
3. The weighted covariance between $H_{S}^{a}$ and $H_{S}^{b}$ is:
  $$C_{\omega }(H_{S}^{a},H_{S}^{b})=\frac{1}{n}\sum_{i=1}^{n}(\omega_{i}{\bar{h}}^{ia}-{\bar{H}}_{S}^{a})(\omega_{i}{\bar{h}}^{ib}-{\bar{H}}_{S}^{b})$$
4. The weighted correlation coefficient between $H_{S}^{a}$ and $H_{S}^{b}$ is:
  $$\begin{array}{c} \rho_{\omega }\left(H_{S}^{a},H_{S}^{b}\right)=\frac{\sum_{i=1}^{n}(\omega_{i}{\bar{h}}^{ai}-{\bar{H}}_{S}^{a})(\omega_{i}{\bar{h}}^{bi}-{\bar{H}}_{S}^{b})}{\sqrt{\sum_{i=1}^{n}{\left(\omega_{i}{\bar{h}}^{ai}-{\bar{H}}_{S}^{a}\right)}^{2}}\sqrt{\sum_{i=1}^{n}{\left(\omega_{i}{\bar{h}}^{bi}-{\bar{H}}_{S}^{b}\right)}^{2}}} \end{array}$$ (7)

### 3.4 Important properties

Now we need to verify that $\rho_{\omega }\left(H_{S}^{a},H_{S}^{b}\right)$ satisfies the following properties.

(i) $C_{\omega }\left(H_{S}^{a},H_{S}^{a}\right)=Var_{\omega }\left(H_{S}^{a}\right)$
(ii) $C_{\omega }\left(H_{S}^{a},H_{S}^{b}\right)=C_{\omega }\left(H_{S}^{b},H_{S}^{a}\right)$
(iii) $C_{\omega }{\left(H_{S}^{a},H_{S}^{b}\right)}^{2}\leq \sqrt{Var_{\omega }\left(H_{S}^{a}\right)}\sqrt{Var_{\omega }\left(H_{S}^{b}\right)}$
(iv) $\rho_{\omega }\left(H_{S}^{a},H_{S}^{b}\right)=\rho_{\omega }\left(H_{S}^{b},H_{S}^{a}\right)$
(v) $\rho_{\omega }\left(H_{S}^{a},H_{S}^{a}\right)=1$
(vi) $-1\leq \rho_{\omega }\left(H_{S}^{a},H_{S}^{b}\right)\leq 1$
(vii) If $H_{S}^{a}=cH_{S}^{b},$ then $\rho_{\omega }\left(H_{S}^{a},H_{S}^{b}\right)=\{\begin{array}{cc} 1; & c>0\\ -1; & c<0 \end{array}$

**Proof (i)** $C_{\omega }\left(H_{S}^{a},H_{S}^{a}\right)$ $=Var_{\omega }\left(H_{S}^{a}\right)$
  $$C_{\omega }(H_{S}^{a},H_{S}^{a})=\frac{1}{n}\sum_{i=1}^{n}(\omega_{i}{\bar{h}}^{ai}-{\bar{H}}_{S}^{a})(\omega_{i}{\bar{h}}^{ai}-{\bar{H}}_{S}^{a})$$
  $$C_{\omega }(H_{S}^{a},H_{S}^{a})=\frac{1}{n}\sum_{i=1}^{n}{\left(\omega_{i}{\bar{h}}^{ai}-{\bar{H}}_{S}^{a}\right)}^{2}=Var_{\omega }\left(H_{S}^{a}\right)$$
**Proof (ii)** is obvious, so we omit the proof.
**Proof (iii)** $C_{\omega }{\left(H_{S}^{a},H_{S}^{b}\right)}^{2}\leq \sqrt{Var_{\omega }\left(H_{S}^{a}\right)}\sqrt{Var_{\omega }\left(H_{S}^{b}\right)}$
  $$C_{\omega }(H_{S}^{a},H_{S}^{b})=\frac{1}{n}\sum_{i=1}^{n}(\omega_{i}{\bar{h}}^{ai}-{\bar{H}}_{S}^{a})(\omega_{i}{\bar{h}}^{bi}-{\bar{H}}_{S}^{b})$$
  $$C_{\omega }(H_{S}^{a},H_{S}^{b})=[\left(\omega_{1}{\bar{h}}^{a1}-{\bar{H}}_{S}^{a}\right)...\left(\omega_{n}{\bar{h}}^{an}-{\bar{H}}_{S}^{a}\right)][\left(\omega_{1}{\bar{h}}^{b1}-{\bar{H}}_{S}^{b}\right)...\left(\omega_{n}{\bar{h}}^{bn}-{\bar{H}}_{S}^{b}\right)]$$
  $$C_{\omega }(H_{s}^{a},H_{s}^{b})={\left|\begin{array}{c} {}[(\left(\omega_{1}{\bar{h}}^{a1}-{\bar{H}}_{S}^{a}\right)\left(\omega_{1}{\bar{h}}^{a1}-{\bar{H}}_{S}^{a}\right)+...+\left(\omega_{n}{\bar{h}}^{an}-{\bar{H}}_{S}^{a}\right)\left(\omega_{n}{\bar{h}}^{an}-{\bar{H}}_{S}^{a}\right)]*\\ \left[\left(\omega_{1}{\bar{h}}^{b1}-{\bar{H}}_{S}^{b}\right)\left(\omega_{1}{\bar{h}}^{b1}-{\bar{H}}_{S}^{b}\right)+...+\left(\omega_{n}{\bar{h}}^{bn}-{\bar{H}}_{S}^{b}\right)\left(\omega_{n}{\bar{h}}^{bn}-{\bar{H}}_{S}^{b}\right)\right] \end{array}\right|}^{1/2}$$
  According to Cauchy-Schwartz inequality
$$C_{\omega }{\left(H_{S}^{a},H_{S}^{b}\right)}^{2}\leq {\left|\begin{array}{c} {}[{\left(\omega_{1}{\bar{h}}^{a1}-{\bar{H}}_{S}^{a}\right)}^{2}+,...,+{\left(\omega_{n}{\bar{h}}^{an}-{\bar{H}}_{S}^{a}\right)}^{2}]*\\ \left[{\left(\omega_{1}{\bar{h}}^{b1}-{\bar{H}}_{S}^{b}\right)}^{2}+,...,+({\left(\omega_{n}{\bar{h}}^{bn}-{\bar{H}}_{S}^{b}\right)}^{2}\right] \end{array}\right|}^{1/2}$$
$$C_{\omega }{\left(H_{S}^{a},H_{S}^{b}\right)}^{2}\leq \sqrt{\frac{1}{n}\sum_{i=1}^{n}{\left(\omega_{i}{\bar{h}}^{ai}-{\bar{H}}_{S}^{a}\right)}^{2}}\sqrt{\frac{1}{n}\sum_{i=1}^{n}{\left(\omega_{i}{\bar{h}}^{bi}-{\bar{H}}_{S}^{b}\right)}^{2}}=\sqrt{Var_{\omega }\left(H_{S}^{a}\right)}\sqrt{Var_{\omega }\left(H_{S}^{b}\right)}$$
**Proof (iv)** is obvious, so we omit the proof.
**Proof (v)** $\rho_{\omega }\left(H_{S}^{a},H_{S}^{a}\right)=1$
$$\rho_{\omega }\left(H_{S}^{a},H_{S}^{a}\right)=\frac{\sum_{i=1}^{n}(\omega_{i}{\bar{h}}^{ai}-{\bar{H}}_{S}^{a})(\omega_{i}{\bar{h}}^{ai}-{\bar{H}}_{S}^{a})}{\sqrt{\sum_{i=1}^{n}{\left(\omega_{i}{\bar{h}}^{ai}-{\bar{H}}_{S}^{a}\right)}^{2}}\sqrt{\sum_{i=1}^{n}{\left(\omega_{i}{\bar{h}}^{ai}-{\bar{H}}_{S}^{a}\right)}^{2}}}$$
$$\rho_{\omega }\left(H_{S}^{a},H_{S}^{a}\right)=\frac{\sum_{i=1}^{n}{\left(\omega_{i}{\bar{h}}^{ai}-{\bar{H}}_{S}^{a}\right)}^{2}}{\sum_{i=1}^{n}{\left(\omega_{i}{\bar{h}}^{ai}-{\bar{H}}_{S}^{a}\right)}^{2}}=1$$
**Proof (vi)** $-1\leq \rho_{\omega }\left(H_{S}^{a},H_{S}^{b}\right)\leq 1$
  The most important property of the correlation coefficient lies between -1 and 1, inclusive. We will prove this property for the special case where we have random variables with zero means and unit variances.
  To prove this property we standardize the two variables as:
  Let
  $$H_{S}^{a}=\frac{\left(\omega_{i}{\bar{h}}^{ai}-\;{\bar{H}}_{S}^{a}\right)}{\sqrt{\sum_{i=1}^{n}{\left(\omega_{i}{\bar{h}}^{ai}-\;{\bar{H}}_{S}^{a}\right)}^{2}}}\;\text{and}\;H_{S}^{b}=\frac{\left(\omega_{i}{\bar{h}}^{bi}-\;{\bar{H}}_{S}^{b}\right)}{\sqrt{\sum_{i=1}^{n}{\left(\omega_{i}{\bar{h}}^{bi}-\;{\bar{H}}_{S}^{b}\right)}^{2}}}$$
  It is obvious that the expected values or means and variances of the two variables are 0 and 1, respectively.
  Symbolically, we write
  $$E(H_{S}^{a})=E(H_{S}^{b})=0\;\text{and}\;Var_{\omega }\left(H_{S}^{a}\right)=Var_{\omega }\left(H_{S}^{b}\right)=1.$$
  We also know that
  $$Var_{\omega }\left(H_{S}^{a}\pm H_{S}^{b}\right)=Var_{\omega }\left(H_{S}^{a}\right)+Var_{\omega }\left(H_{S}^{b}\right)\pm 2C_{\omega }\left(H_{S}^{a},H_{S}^{b}\right)$$
  But $C_{\omega }\left(H_{S}^{a},H_{S}^{b}\right)=E\left(H_{S}^{a}H_{S}^{b}\right)-E\left(H_{S}^{a}\right)E\left(H_{S}^{b}\right)=E\left(H_{S}^{a}H_{S}^{b}\right)$

$\left(∵E\left(H_{S}^{a}\right)=E\left(H_{S}^{b}\right)=0\right)$
$$\Rightarrow E[\frac{\left(\omega_{i}h^{a}-E\left(H_{S}^{a}\right))(\omega_{i}h^{b}-E\left(H_{S}^{b}\right)\right)}{\sqrt{Var_{\omega }\left(H_{S}^{b}\right)Var_{\omega }\left(H_{S}^{b}\right)}}]=\rho_{\omega }$$
$$=\frac{\sum_{i=1}^{n}(\omega_{i}{\bar{h}}^{ai}-{\bar{H}}_{S}^{a})(\omega_{i}{\bar{h}}^{bi}-{\bar{H}}_{S}^{b})}{\sqrt{\sum_{i=1}^{n}{\left(\omega_{i}{\bar{h}}^{ai}-{\bar{H}}_{S}^{a}\right)}^{2}}\sqrt{\sum_{i=1}^{n}{\left(\omega_{i}{\bar{h}}^{bi}-{\bar{H}}_{S}^{b}\right)}^{2}}}$$
  Thus $Var_{\omega }\left(H_{S}^{a}+H_{S}^{b}\right)=1+1+2\rho_{\omega }$
  $$\begin{array}{c} =1+1+2[\frac{\sum_{i=1}^{n}(\omega_{i}{\bar{h}}^{ai}-{\bar{H}}_{S}^{a})(\omega_{i}{\bar{h}}^{bi}-{\bar{H}}_{S}^{b})}{\sqrt{\sum_{i=1}^{n}{\left(\omega_{i}{\bar{h}}^{ai}-{\bar{H}}_{S}^{a}\right)}^{2}}\sqrt{\sum_{i=1}^{n}{\left(\omega_{i}{\bar{h}}^{bi}-{\bar{H}}_{S}^{b}\right)}^{2}}}]\\ =\;2(1+\rho_{\omega })\\ =2(1+\frac{\sum_{i=1}^{n}(\omega_{i}{\bar{h}}^{ai}-{\bar{H}}_{S}^{a})(\omega_{i}{\bar{h}}^{bi}-{\bar{H}}_{S}^{b})}{\sqrt{\sum_{i=1}^{n}{\left(\omega_{i}{\bar{h}}^{ai}-{\bar{H}}_{S}^{a}\right)}^{2}}\sqrt{\sum_{i=1}^{n}{\left(\omega_{i}{\bar{h}}^{bi}-{\bar{H}}_{S}^{b}\right)}^{2}}}) \end{array}$$
  Since $Var_{\omega }\left(H_{S}^{a}+H_{S}^{b}\right)$ must be non-negative, therefore it follows that 2(1+ *ρ*_*ω*_)
$$\Rightarrow 2(1+\frac{\sum_{i=1}^{n}(\omega_{i}{\bar{h}}^{ai}-{\bar{H}}_{S}^{a})(\omega_{i}{\bar{h}}^{bi}-{\bar{H}}_{S}^{b})}{\sqrt{\sum_{i=1}^{n}{\left(\omega_{i}{\bar{h}}^{ai}-{\bar{H}}_{S}^{a}\right)}^{2}}\sqrt{\sum_{i=1}^{n}{\left(\omega_{i}{\bar{h}}^{bi}-{\bar{H}}_{S}^{b}\right)}^{2}}})\geq 0$$
$$\Rightarrow \frac{\sum_{i=1}^{n}(\omega_{i}{\bar{h}}^{ai}-{\bar{H}}_{S}^{a})(\omega_{i}{\bar{h}}^{bi}-{\bar{H}}_{S}^{b})}{\sqrt{\sum_{i=1}^{n}{\left(\omega_{i}{\bar{h}}^{ai}-{\bar{H}}_{S}^{a}\right)}^{2}}\sqrt{\sum_{i=1}^{n}{\left(\omega_{i}{\bar{h}}^{bi}-{\bar{H}}_{S}^{b}\right)}^{2}}}\geq -1$$
  Similarly $Var_{\omega }\left(H_{S}^{a}-H_{S}^{b}\right)=2\left(1-\rho_{\omega }\right)$
  $$=2(1-\frac{\sum_{i=1}^{n}(\omega_{i}{\bar{h}}^{ai}-{\bar{H}}_{S}^{a})(\omega_{i}{\bar{h}}^{bi}-{\bar{H}}_{S}^{b})}{\sqrt{\sum_{i=1}^{n}{\left(\omega_{i}{\bar{h}}^{ai}-{\bar{H}}_{S}^{a}\right)}^{2}}\sqrt{\sum_{i=1}^{n}{\left(\omega_{i}{\bar{h}}^{bi}-{\bar{H}}_{S}^{b}\right)}^{2}}})$$
  $$\Rightarrow \frac{\sum_{i=1}^{n}(\omega_{i}{\bar{h}}^{ai}-{\bar{H}}_{S}^{a})(\omega_{i}{\bar{h}}^{bi}-{\bar{H}}_{S}^{b})}{\sqrt{\sum_{i=1}^{n}{\left(\omega_{i}{\bar{h}}^{ai}-{\bar{H}}_{S}^{a}\right)}^{2}}\sqrt{\sum_{i=1}^{n}{\left(\omega_{i}{\bar{h}}^{bi}-{\bar{H}}_{S}^{b}\right)}^{2}}}\leq 1.$$
  Hence from these two results, we get
  $$=-1\leq \frac{\sum_{i=1}^{n}(\omega_{i}{\bar{h}}^{ai}-{\bar{H}}_{S}^{a})(\omega_{i}{\bar{h}}^{bi}-{\bar{H}}_{S}^{b})}{\sqrt{\sum_{i=1}^{n}{\left(\omega_{i}{\bar{h}}^{ai}-{\bar{H}}_{S}^{a}\right)}^{2}}\sqrt{\sum_{i=1}^{n}{\left(\omega_{i}{\bar{h}}^{bi}-{\bar{H}}_{S}^{b}\right)}^{2}}}\leq 1$$
  $$\Rightarrow -1\leq \rho_{\omega }\left(H_{S}^{a},H_{S}^{b}\right)\leq 1.$$
**Proof (vii)** Let $H_{S}^{a}=cH_{S}^{b},$ then for *c* > 0
$$\rho_{\omega }=\frac{\sum_{i=1}^{n}(c\omega_{i}{\bar{h}}^{bi}-c{\bar{H}}_{S}^{b})(\omega_{i}{\bar{h}}^{bi}-{\bar{H}}_{S}^{b})}{\sqrt{\sum_{i=1}^{n}{\left(c\omega_{i}{\bar{h}}^{bi}-c{\bar{H}}_{S}^{b}\right)}^{2}}\sqrt{\sum_{i=1}^{n}{\left(\omega_{i}{\bar{h}}^{bi}-{\bar{H}}_{S}^{b}\right)}^{2}}}=\frac{c\sum_{i=1}^{n}{\left(\omega_{i}{\bar{h}}^{bi}-{\bar{H}}_{S}^{b}\right)}^{2}}{\sqrt{c^{2}\sum_{i=1}^{n}{\left(\omega_{i}{\bar{h}}^{bi}-{\bar{H}}_{S}^{b}\right)}^{2}}}=1$$
$$\Rightarrow \rho_{\omega }\left(H_{S}^{a},H_{S}^{b}\right)=1$$
  Similarly, for *c* < 0, $\rho_{\omega }\left(H_{S}^{a},H_{S}^{b}\right)=-1$
  Hence $\rho_{\omega }\left(H_{S}^{a},H_{S}^{b}\right)=\{\begin{array}{cc} 1;\;\;\; & \;c>0\\ -1; & \;c<0 \end{array}$

### 3.5 Ordered weighted correlation coefficient of H2FLSs

We first rank the alternatives in ordered weighted correlation measures and afterwards weights them regarding their ranking positions. The ordered weighted correlation has been widely examined in [51]. Here, we discuss the ordered weighted correlation measure between H2FLSs. Suppose that the weight associated with each position is *γ*_*i*_, where *γ*_*i*_ ∈ [0, 1] (*i* = 1, 2, …, *n*) and $\sum_{i=1}^{n}\gamma_{i}=1.$ Then, the understudy idea identified with the ordered weighted correlation measure can be presented as:

1. The ordered weighted mean of the H2FLS is:
  $${\bar{H}}_{S(\gamma )}=\frac{1}{n}\sum_{i=1}^{n}(\gamma_{i}{\bar{h}}^{i})$$
2. The ordered weighted variance of the H2FLS is:
  $$Var_{\gamma }\left(H_{S}\right)=\frac{1}{n}\sum_{i=1}^{n}{\left(\gamma_{i}{\bar{h}}^{i}-{\bar{H}}_{s}\right)}^{2}$$
3. The ordered weighted covariance between $H_{S}^{a}$ and $H_{S}^{b}$ is:
  $$C_{\gamma }(H_{S}^{a},H_{S}^{b})=\frac{1}{n}\sum_{i=1}^{n}(\gamma_{i}{\bar{h}}^{ia}-{\bar{H}}_{S}^{a})(\gamma_{i}{\bar{h}}^{ib}-{\bar{H}}_{S}^{b})$$
4. The ordered weighted correlation coefficient between $H_{S}^{a}$ and $H_{S}^{b}$ is:
  $$\begin{array}{c} \rho_{\gamma }\left(H_{S}^{a},H_{S}^{b}\right)=\frac{\sum_{i=1}^{n}(\gamma_{i}{\bar{h}}^{ai}-{\bar{H}}_{S}^{a})(\gamma_{i}{\bar{h}}^{bi}-{\bar{H}}_{S}^{b})}{\sqrt{\sum_{i=1}^{n}{\left(\gamma_{i}{\bar{h}}^{ai}-{\bar{H}}_{S}^{a}\right)}^{2}}\sqrt{\sum_{i=1}^{n}{\left(\gamma_{i}{\bar{h}}^{bi}-{\bar{H}}_{S}^{b}\right)}^{2}}} \end{array}$$ (8)

## 4 Method to determine the criteria weights

**Fig 1:**
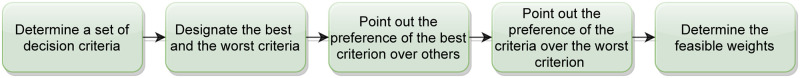
The procedure of BWM used to obtain the weights of criteria.

Experts have weighed the measurements in some circumstances, but sometimes it just does not. For the case, we must determine the weight of each criterion where the weight vector is not exact. In a situation where there is no evidence to confirm differences in criteria, we can assume that all criteria are considered equal. Otherwise, we must face a strategy to determine the weight of the criteria. We should handle the strategy to measure the criteria weights in the position where there is no evidence to confirm the difference in patterns. This part applies BWM to solve this issue. Here, we shortly discuss the procedure of BWM that can be utilized to obtain the weights of criteria [52]. Fig 1 shows summary of this procedure.

**Step 1.** Determine a set of decision criteria: In this progression, the experts recognizes *n* criteria {*C*_1_, *C*_2_, …, *C*_*n*_} that are utilized to create a decision.
**Step 2.** Decide the best (for example most attractive, most significant) and the worst (for example least attractive, least significant) criteria.
**Step 3.** Decide the preference of the best criteria over all the others, H2FLEs of best criterion to all the other criteria is performed and then transformed to numbers according to (1). The consequent best-to-other vector would be: *Q*_*B*_ = (*q*_*B*1_, *q*_*B*2_, …, *q*_*Bn*_), where *q*_*Bj*_ shows the preference of the best criterion B over criterion j. Obviously *q*_*BB*_ = 1
**Step 4.** Decide the preference of the considerable number of criteria over the worst criteria, utilizing H2FLEs and then transformed to numbers according to (1). The consequent others-to worst vector would be: *Q*_*W*_ = (*q*_1*B*_, *q*_2*B*_, …, *q*_*nB*_)^*T*^, where *q*_*jW*_ Shows the preference of the criterion j over the worst criterion W. Obviously *q*_*WW*_ = 1
**Step 5.** Determine the feasible weights $\left(P_{w1}^{*},P_{w2}^{*},...,P_{wn}^{*}\right)$ [52].

To derive the above weights presented in Step 5, we minimize the maximum among the set of {|*P*_*wB*_ − *q*_*Bj*_*P*_*wj*_|, |*P*_*wj*_ − *q*_*jW*_*P*_*wW*_|}. Hence it can be formulated as follows:

$$\text{min}\underset{j}{\text{max}}\{|P_{wB}-q_{Bj}P_{wj}|,|P_{wj}-q_{jW}P_{wW}|\}$$

subject to

$$\begin{array}{c} \sum P_{wj}=1 \end{array}$$ (9)

$P_{wj}\;≽\;0,$ for all *j*.

Model (9) can be transferred to the following linear programming model:

$$min\zeta^{L}$$

subject to

$$\begin{array}{c} |P_{wB}-q_{Bj}P_{wj}|\leq \zeta^{L},\;\text{for}\;\text{all}\;j\\ |P_{wj}-q_{jW}P_{wW}|\leq \zeta^{L},\;\text{for}\;\text{all}\;j\\ \sum P_{wj}=1 \end{array}$$ (10)

$P_{wj}\;≽\;0,$ for all *j*

By solving problem (10), the optimal weights $\left(P_{w1}^{*},P_{w2}^{*},...,P_{wn}^{*}\right)$ and *ζ*^*L*^ are obtained.

### 4.1 MCGDM problem solving approach based on the proposed correlation coefficient for H2FLSs

**Fig 2:**
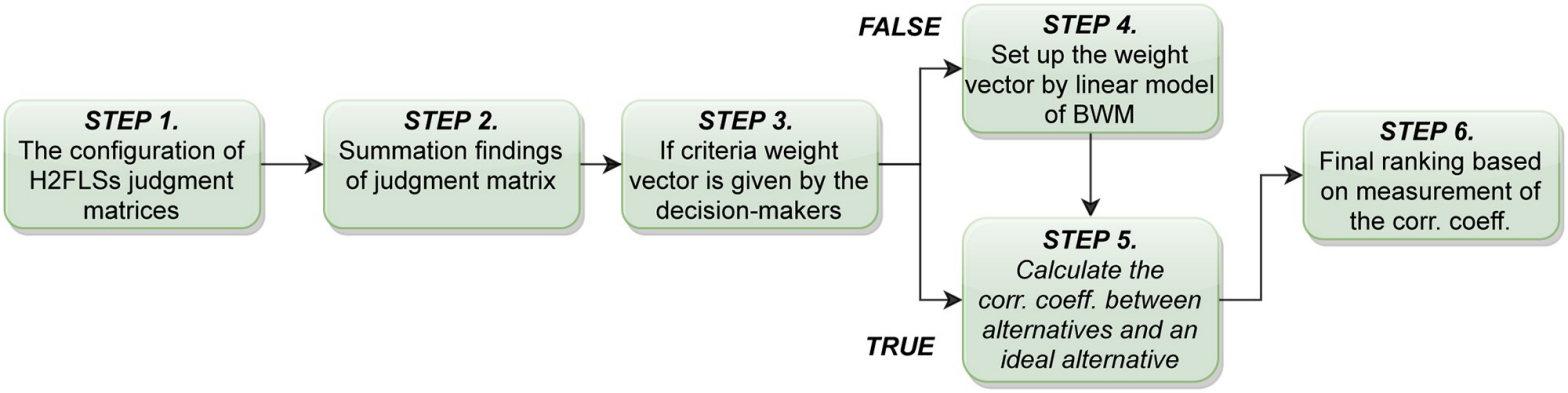
An algorithm for solving the problem using the proposed corr. coeff.

In the current subsection, a method of MCGDM for decision-making purposes in the environment of H2FLSs is analyzed. Assume, there is a set of *n* alternatives *A* = {*A*_1_, *A*_2_, …, *A*_*n*_}, where alternatives are assessed under *m* criteria, represented by *C* = {*C*_1_, *C*_2_, …, *C*_*m*_}. The H2FLSs estimation of *A*_*i*_ under *C*_*j*_ is represented by *A*_*ij*_. Let, there is a set of *k* experts *D* = {*D*_1_, *D*_2_, …, *D*_*K*_} and they are proposed to keep confidential their conclusions to get a progressively exact outcome. The criterion weight *C*_*j*_ is *w*_*j*_, where *w*_*j*_ ≥ 0 (*j* = 1, 2, …, *m*) and *Σw*_*j*_ = 1. The decision among different alternatives contain the steps which are summarized in the Fig 2.

**Step 1.** The configuration of H2FLSs judgment matrices *R*(*t*) (*t* = 1, …, *K*) established in the authoritative perspective on the efficiency of *A*_*i*_ (*i* = 1, …, *n*) on criteria *C*_*j*_ (*j* = 1, …, *m*) using the LTS *S*. (see [24]).
**Step 2.** The configuration of H2FLSs as summation findings of matrix *R*. (see [24]).
**Step 3.** Check out even if the weight vector of criteria is presented by the decision-makers or not. If present, proceed to Step 5. If not, go to the next step.
**Step 4.** Set up the weight vector by linear model of BWM [52] (section 4), proceed to the next step.
**Step 5.** Computation of correlation coefficient between different alternatives with an ideal alternative. An ideal alternative has the maximum membership degree and minimum non-membership degree.
**Step 6.** Ranking of the alternatives on the basis of measurement of the correlation coefficient. The best alternative has the largest correlation coefficient.

## 5 Numerical example (with known & unknown criteria weights)

To feature the practicality and viability of the planned decision-making method, two models are utilized from some relevant literature [24, 53, 54].

Assume an organization needs to pick a provider with the most grounded far quality for improving its administration levels. There involve four suppliers *A*_*i*_ = {*A*_1_, *A*_2_, *A*_3_, *A*_4_}. Three assessment criteria are considered for four goods providers, i.e., quality (*C*_1_), supply capacity and flexibility (*C*_2_), and price (*C*_3_) with *ω* = (0.504, 0.335, 0.161). Experts give their views based on the LTS, S = {*u*_0_ =nothing, *u*_1_ = very poor, *u*_2_ = poor, *u*_3_ = moderate, *u*_4_ = good, *u*_5_ = very good, *u*_6_ = perfect}.

The H2FLSs aggregated judgment matrix is used the same as obtained in Wang et al. [24], given as follows:

**Table 1:** H2FLSs aggregated judgment matrix.

Alternatives	*C*_1_	*C*_2_	*C*_3_
*A*_1_	((*u*_1_, 0.0467), (*u*_1_, 0.1656))	((*u*_1_, −0.2254))	((*u*_1_, −0.0926), (*u*_1_, −0.0544), (*u*_1_, 0.0127), (*u*_1_, 0.0557))
*A*_2_	((*u*_1_, −0.3717), (*u*_1_, −0.2540))	((*u*_1_, 0.0997), (*u*_1_, 0.2012), (*u*_1_, 0.2055), (*u*_1_, 0.3197))	((*u*_1_, −0.2254))
*A*_3_	((*u*_0_, 0.4899))	((*u*_1_, 0.0498), (*u*_1_, 0.0758))	((*u*_1_, −0.2082), (*u*_1_, −0.1735), (*u*_1_, −0.1099), (*u*_1_, −0.0926), (*u*_1_, −0.0767), (*u*_1_, −0.0544), (*u*_1_, 0.0197), (*u*_1_, 0.0577))
*A*_4_	((*u*_1_, 0.1457), (*u*_1_, 0.1660), (*u*_1_, 0.2520) (*u*_1_, 0.2737))	((*u*_1_, 0.0081), (*u*_1_, 0.0903))	((*u*_1_, −0.2254))

**Step 1 & 2.** Taking an ideal alternative for the calculation of correlation coefficients between the alternatives of Table 1 and ideal alternative. The ideal alternative for the given example is as follows:

$$A^{*}=\left\{\left(\left(u_{3},0.5\right),\left(u_{2},0.25\right)\right)\left(\left(u_{3},0.15\right),\left(u_{2},0.15\right)\right)\left(\left(u_{3},0.15\right),\left(u_{2},0.5\right)\right)\right\}$$

**Step 3 & 4.** Weight vector is given by the decision-makers, so we omit the steps.

**Step 5.** The correlation coefficients of each alternative *A*_*i*_ (1 ≤ *i* ≤ 4) and A* with respect to their given weight are calculated as follows in Table 2.

**Table 2:** Calculation results with respect to the given weights.

Alternatives	$\omega^{i}{\bar{a}}^{1}$	$\omega^{i}{\bar{a}}^{2}$	$\omega^{i}{\bar{a}}^{3}$	${\bar{A}}_{i}$	*Var*_*ω*_(*A*_*i*_)	*C*_*ω*_(*A*_*i*_, A*)	*ρ*_*ω*_(*A*_*i*_, A*)
*A*_1_	0.5575	0.2595	0.1578	0.3249	0.0863	0.2029	0.9797
*A*_2_	0.3463	0.4042	0.1247	0.2917	0.0435	0.1029	0.7000
*A*_3_	0.2469	0.3560	0.1482	0.2504	0.0216	0.0423	0.4081
*A*_4_	0.6095	0.3515	0.1247	0.3619	0.1177	0.2417	0.9993
A*	1.4490	0.8878	0.4548	0.9305	0.4969	0.4969	1.0000

**Step 6.** In response to the estimations of correlation coefficients in step 3, the alternatives are ranked. The ranking order is acquired as *A*_4_ > *A*_1_ > *A*_2_ > *A*_3_. Consequently, The organization will pick *A*_4_ for their services.

In the given an example, it is seen that the weight vector is presented already by the decision-makers, but sometimes, it is likely that the weight vector is obscure. If so, we can utilize the BWM to determine the weights of the criteria. Here we again go back to step 4 only to show what to do if we lack this knowledge and set up the weight vector.

**Step 4.** The company recognizes Quality (*C*_1_) for best and Price (*C*_3_) for the worst criteria. Tables 3 and 4 demonstrate the comparison vectors. Due to the ambiguity and intangibility of decision-making, H2FLSs is used for optimal company selection.

**Table 3:** Pairwise comparison vector for the best criterion.

Criteria	*C*_1_	*C*_2_	*C*_3_
Best criterion: *C*_1_	((*u*_1_, 0), (*u*_1_, 0))	((*u*_4_, −0.2254), (*u*_2_, 0.2144))	((*u*_5_, −0.2134), (*u*_3_, 0.1234))

**Table 4:** Pairwise comparison vector for the worst criterion.

Criteria	Worst Criterion: *C*_3_
*C*_1_	((*u*_5_, −0.2134), (*u*_3_, 0.1234))
*C*_2_	((*u*_2_, 0.2024), (*u*_1_, 0.2554))
*C*_3_	((*u*_1_, 0), (*u*_1_, 0))

After the simplification of the given data presented in Tables 3 and 4 by using (1), we have the following results in the model (10) for this problem, as follows:

min *ζ*^*L*^

subject to

$$|P_{w1}-2.9945P_{w2}|\leq \zeta^{L}$$

$$|P_{w1}-3.9550P_{w3}|\leq \zeta^{L}$$

$$|P_{w2}-1.7289P_{w3}|\leq \zeta^{L}$$

$$P_{w1}+P_{w2}+P_{w3}=1$$

$$P_{w1},P_{w2},P_{w3}\geq 0$$

Solving this model, we have (stage 5): $P_{w1}^{*}=0.628,$ $P_{w2}^{*}=0.222$ and $P_{w3}^{*}=0.150.$

**Step 5.** The correlation coefficients of each alternative *A*_*i*_ (1 ≤ *i* ≤ 4) and A* with respect to their weights are calculated as follows:

**Table 5:** Calculation results with respect to the BWM.

Alternatives	$\;\omega^{i}{\bar{a}}^{1}$	$\;\omega^{i}{\bar{a}}^{2}$	$\;\omega^{i}{\bar{a}}^{3}$	$\;\;{\bar{A}}_{i}$	*Var*_*ω*_(*A*_*i*_)	*C*_*ω*_(*A*_*i*_, A*)	*ρ*_*ω*_(*A*_*i*_, A*)
*A*_1_	0.6947	0.1720	0.1471	0.3379	0.1912	0.4656	0.9976
*A*_2_	0.4315	0.2678	0.1162	0.2719	0.0497	0.2200	0.9240
*A*_3_	0.3077	0.2359	0.1380	0.2272	0.0145	0.1126	0.8760
*A*_4_	0.7595	0.2329	0.1162	0.3695	0.2349	0.5163	0.9981
A*	1.8055	0.5883	0.4238	0.9392	1.1393	1.1393	1.0000

**Table 6:** Calculation results of ordered weighted correlation coefficients with respect to the BWM.

Alternatives	$\;\omega^{i}{\bar{a}}^{1}$	$\;\omega^{i}{\bar{a}}^{2}$	$\;\omega^{i}{\bar{a}}^{3}$	$\;\;{\bar{A}}_{i}$	$Var_{\gamma }\left(A_{i}\right)$	$C_{\gamma }\left(A_{i},A^{*}\right)$	$\rho_{\gamma }\left(A_{i},A^{*}\right)$
*A*_1_	0.6947	0.2176	0.1162	0.3428	0.0382	0.0933	0.9999
*A*_2_	0.7577	0.1720	0.1031	0.3442	0.0518	0.1085	0.9984
*A*_3_	0.6674	0.2043	0.0735	0.3151	0.0390	0.0941	0.9983
*A*_4_	0.7598	0.2330	0.1162	0.3695	0.0470	0.1035	0.9998
A*	1.8055	0.6272	0.3975	0.9434	0.2282	0.2282	1.0000

**Step 6.** As per the estimations of correlation coefficients in Tables 5 & 6, we come to know that the *ρ*_*ω*_(*A*_4_, A*) in Table 5 and $\rho_{\gamma }\left(A_{1},A^{*}\right)$ in Table 6, has the maximum correlation.

## 6 Comparative analysis

The area of study being talked about here keep up a comparative study among the existing methods proposed by [24, 52, 53] with our work. Referring to Table 7 of the above example, the ranking order of the alternatives is as follows:

**Table 7:** Comparison of obtained results.

Researchers	Ranking	Optimal alternative
Wang et al. [24]	*A*_1_ > *A*_4_ > *A*_2_ > *A*_3_	*A*_1_
Ge & Wei [53]	*A*_4_ > *A*_1_ > *A*_2_ > *A*_3_	*A*_4_
Wang et al. [54]	*A*_4_ > *A*_1_ > *A*_2_ > *A*_3_	*A*_4_
Our proposed method ranking w.r.t given weights	*A*_4_ > *A*_1_ > *A*_2_ >*A*_3_	*A*_4_
Our proposed method ranking w.r.t BWM criteria	*A*_4_ > *A*_1_ > *A*_2_ >*A*_3_	*A*_4_
Ordered weighted correlation coefficient ranking	*A*_1_ > *A*_4_ > *A*_2_ >*A*_3_	*A*_1_

Wang et al. [24] dealt with a similar example utilizing hesitant 2-tuple linguistic Bonferroni mean operator and prioritized weighted hesitant 2-tuple linguistic Bonferroni mean operator, in which the prioritization relationship of specialists is considered. By analyzing the two ranking outcomes in Table 7, the proposed order weighted correlation, and Wang et al. method are similar. However, there exists a difference in positioning consequence of the first two alternatives *A*_1_ and *A*_4_ of the proposed weighted correlation with wang et al. alternatives ranking order. The reason is that the different prioritization relationships of experts considered by Wang et al. can cause changes in overall values. However, we can say that the overall ranking order is stable and adequate.

Ge & Wei [53] also coped with a similar example using hesitant 2-tuple weighted averaging operator and hesitant 2-tuple ordered weighted averaging operator, in which the prioritization relationship of specialists is not thought of, and criteria weights are given in advance. Wang et al. [54] likewise proposed a technique to deal with a similar illustration by utilizing hesitant 2-tuple linguistic prioritized weighted averaging operator and hesitant 2-tuple linguistic correlated averaging operator. In [53, 54] and our proposed approach, the most feasible option is *A*_4_, which indicates the validity of the proposed work.

Similarly, it can also be seen that the ranking order obtained from the proposed method using BWM criteria weights is very similar to the ranking order of alternatives obtained in [24, 53, 54]. From the above comparisons, the obtained ranking orders of the alternatives show the efficiency of the proposed correlation coefficient. The proposed approach can be applied in every field where there is a relationship between two variables. Furthermore, the proposed approach is easy to calculate for H2FLSs compared to other methodologies mentioned in section 1, which are based on different approaches. As a result, we can conclude that our work is strong and capable of solving the MCGDM problem in the context of H2FLSs compared to the existing approaches’ computational difficulties.

## 7 Conclusion

The correlation measure of H2FLSs investigated in this paper, which lies in the [−1, 1], gives us more information than that the correlation presented by [26]. The presented correlation coefficient in the context of the hesitant 2-tuple fuzzy linguistic environment indicates the degree of relationship between two variables and investigates their linear change in a positive or negative direction. We first explored the ideas of the mean and variance of H2FLEs, and afterwards proposed a new correlation coefficient. Linked to current methodologies, the most important feature of the proposed method identifies the positive and negative associations for H2FLSs. We additionally defined the weighted and ordered weighted correlation coefficients of the H2FLSs. We have proved some desired properties for the covariance and the correlation coefficient. BWM has also been used to determine the weights of criteria and alternatives w.r.t different criteria. The validation of this work is strengthened by the parallel findings of the suggested method with the available ones. The proposed work is appropriate for H2FLSs and easy to calculate, also capable to holds the property of solving MCGDM problems. In the future, we plan to propose the correlation coefficient for intuitionistic H2FLSs and apply them to the MCGDM problems.

## References

[1] Alcalde C, Burusco A, Fuentes-González R, Zubia I. The use of linguistic variables and fuzzy propositions in the L-fuzzy concept theory. Computers & Mathematics with Applications. 2011;62(8):3111–3122.

[2] Parreiras RO, Ekel PY, Martini J, Palhares RM. A flexible consensus scheme for multicriteria group decision making under linguistic assessments. Information Sciences. 2010;180(7):1075–1089. doi: 10.1016/j.ins.2009.11.046

[3] Zadeh LA. Fuzzy sets versus probability. Proceedings of the IEEE. 1980;68(3):421–421. doi: 10.1109/PROC.1980.11659

[4] Cao Qw, Wu J. The extended COWG operators and their application to multiple attributive group decision making problems with interval numbers. Applied Mathematical Modelling. 2011;35(5):2075–2086. doi: 10.1016/j.apm.2010.11.040

[5] Yue Z. An extended TOPSIS for determining weights of decision makers with interval numbers. Knowledge-based systems. 2011;24(1):146–153. doi: 10.1016/j.knosys.2010.07.014

[6] Dong Y, Chen X, Herrera F. Minimizing adjusted simple terms in the consensus reaching process with hesitant linguistic assessments in group decision making. Information Sciences. 2015;297:95–117. doi: 10.1016/j.ins.2014.11.011

[7] Zadeh LA. The concept of a linguistic variable and its application to approximate reasoning. Information sciences. 1975;8(3):199–249. doi: 10.1016/0020-0255(75)90036-5

[8] Liu W, Liao H. A bibliometric analysis of fuzzy decision research during 1970–2015. International Journal of Fuzzy Systems. 2017;19(1):1–14. doi: 10.1007/s40815-016-0272-z

[9] Rodriguez RM, Martinez L, Herrera F. Hesitant fuzzy linguistic term sets for decision making. IEEE Transactions on fuzzy systems. 2011;20(1):109–119. doi: 10.1109/TFUZZ.2011.2170076

[10] Liao H, Xu Z, Herrera-Viedma E, Herrera F. Hesitant fuzzy linguistic term set and its application in decision making: a state-of-the-art survey. International Journal of Fuzzy Systems. 2018;20(7):2084–2110. doi: 10.1007/s40815-018-0561-9

[11] Wang H, Xu Z, Zeng XJ. Hesitant fuzzy linguistic term sets for linguistic decision making: Current developments, issues and challenges. Information Fusion. 2018;43:1–12. doi: 10.1016/j.inffus.2017.11.010

[12] Herrera F, Martínez L. A 2-tuple fuzzy linguistic representation model for computing with words. IEEE Transactions on fuzzy systems. 2000;8(6):746–752. doi: 10.1109/91.890332

[13] Herrera F, Martinez L. An approach for combining linguistic and numerical information based on the 2-tuple fuzzy linguistic representation model in decision-making. International Journal of Uncertainty, Fuzziness and Knowledge-Based Systems. 2000;8(05):539–562. doi: 10.1142/S0218488500000381

[14] Beg I, Rashid T. Hesitant 2-tuple linguistic information in multiple attributes group decision making. Journal of Intelligent & Fuzzy Systems. 2016;30(1):109–116. doi: 10.3233/IFS-151737

[15] Chen Z, Liu P, Pei Z. An approach to multiple attribute group decision making based on linguistic intuitionistic fuzzy numbers. International Journal of Computational Intelligence Systems. 2015;8(4):747–760. doi: 10.1080/18756891.2015.1061394

[16] Martínez L, Rodriguez RM, Herrera F. 2-tuple linguistic model. In: The 2-tuple Linguistic Model. Springer; 2015. p. 23–42.

[17] Martı L, Herrera F, et al. An overview on the 2-tuple linguistic model for computing with words in decision making: Extensions, applications and challenges. Information Sciences. 2012;207:1–18. doi: 10.1016/j.ins.2012.04.025

[18] Rodríguez RM, Martínez L. An analysis of symbolic linguistic computing models in decision making. International Journal of General Systems. 2013;42(1):121–136. doi: 10.1080/03081079.2012.710442

[19] Hu Z, Rao C, Zheng Y, Huang D. Optimization decision of supplier selection in green procurement under the mode of low carbon economy. International Journal of Computational Intelligence Systems. 2015;8(3):407–421. doi: 10.1080/18756891.2015.1017375

[20] Merigo JM, Casanovas M, Martínez L. Linguistic aggregation operators for linguistic decision making based on the Dempster-Shafer theory of evidence. International Journal of Uncertainty, Fuzziness and Knowledge-Based Systems. 2010;18(03):287–304. doi: 10.1142/S0218488510006544

[21] Xue YX, You JX, Zhao X, Liu HC. An integrated linguistic MCDM approach for robot evaluation and selection with incomplete weight information. International Journal of Production Research. 2016;54(18):5452–5467. doi: 10.1080/00207543.2016.1146418

[22] Zhang Y, Ma H, Liu B, Liu J. Group decision making with 2-tuple intuitionistic fuzzy linguistic preference relations. Soft Computing. 2012;16(8):1439–1446. doi: 10.1007/s00500-012-0847-z

[23] Wei C, Liao H. A multigranularity linguistic group decision-making method based on hesitant 2-tuple sets. International Journal of Intelligent Systems. 2016;31(6):612–634. doi: 10.1002/int.21798

[24] Wang L, Wang Y, Pedrycz W. Hesitant 2-tuple linguistic Bonferroni operators and their utilization in group decision making. Applied Soft Computing. 2019;77:653–664. doi: 10.1016/j.asoc.2019.01.038

[25] Hong DH. Fuzzy measures for a correlation coefficient of fuzzy numbers under TW (the weakest t-norm)-based fuzzy arithmetic operations. Information Sciences. 2006;176(2):150–160. doi: 10.1016/j.ins.2004.11.005

[26] Liao H, Xu Z, Zeng XJ, Merigó JM. Qualitative decision making with correlation coefficients of hesitant fuzzy linguistic term sets. Knowledge-Based Systems. 2015;76:127–138. doi: 10.1016/j.knosys.2014.12.009

[27] Saneifard R, Saneifard R. Correlation coefficient between fuzzy numbers based on central interval. Journal of Fuzzy Set Valued Analysis. 2012;2012(March):1–9. doi: 10.5899/2012/jfsva-00051

[28] Chiang DA, Lin NP. Correlation of fuzzy sets. Fuzzy sets and systems. 1999;102(2):221–226. doi: 10.1016/S0165-0114(97)00127-9

[29] Liu ST, Kao C. Fuzzy measures for correlation coefficient of fuzzy numbers. Fuzzy Sets and Systems. 2002;128(2):267–275. doi: 10.1016/S0165-0114(01)00199-3

[30] Zhang R, Li Z, Liao H. Multiple-attribute decision-making method based on the correlation coefficient between dual hesitant fuzzy linguistic term sets. Knowledge-Based Systems. 2018;159:186–192. doi: 10.1016/j.knosys.2018.07.014

[31] Tyagi SK. Correlation coefficient of dual hesitant fuzzy sets and its applications. Applied mathematical modelling. 2015;39(22):7082–7092. doi: 10.1016/j.apm.2015.02.046

[32] Chen N, Xu Z, Xia M. Correlation coefficients of hesitant fuzzy sets and their applications to clustering analysis. Applied Mathematical Modelling. 2013;37(4):2197–2211. doi: 10.1016/j.apm.2012.04.031

[33] Arora R, Garg H. A robust correlation coefficient measure of dual hesitant fuzzy soft sets and their application in decision making. Engineering Applications of Artificial Intelligence. 2018;72:80–92. doi: 10.1016/j.engappai.2018.03.019

[34] Li Y, Wang J, Zhao D, Li G, Chen C. A two-stage approach for combined heat and power economic emission dispatch: Combining multi-objective optimization with integrated decision making. Energy. 2018;162:237–254. doi: 10.1016/j.energy.2018.07.200

[35] Edwards W, Barron FH. SMARTS and SMARTER: Improved simple methods for multiattribute utility measurement. Organizational behavior and human decision processes. 1994;60(3):306–325. doi: 10.1006/obhd.1994.1087

[36] Saaty TL. What is the analytic hierarchy process? In: Mathematical models for decision support. Springer; 1988. p. 109–121. doi: 10.1007/978-3-642-83555-1_5

[37] Saaty TL. How to make a decision: the analytic hierarchy process. European journal of operational research. 1990;48(1):9–26. doi: 10.1016/0377-2217(90)90057-I 11659401

[38] Rezaei J. Best-worst multi-criteria decision-making method. Omega. 2015;53:49–57. doi: 10.1016/j.omega.2014.11.009

[39] Ahmadi HB, Kusi-Sarpong S, Rezaei J. Assessing the social sustainability of supply chains using Best Worst Method. Resources, Conservation and Recycling. 2017;126:99–106. doi: 10.1016/j.resconrec.2017.07.020

[40] Mi X, Tang M, Liao H, Shen W, Lev B. The state-of-the-art survey on integrations and applications of the best worst method in decision making: Why, what, what for and what’s next? Omega. 2019;87:205–225. doi: 10.1016/j.omega.2019.01.009

[41] Mohammadi M, Rezaei J. Bayesian best-worst method: A probabilistic group decision making model. Omega. 2020;96:102075. doi: 10.1016/j.omega.2019.06.001

[42] Brunelli M, Rezaei J. A multiplicative best–worst method for multi-criteria decision making. Operations Research Letters. 2019;47(1):12–15. doi: 10.1016/j.orl.2018.11.008

[43] Hafezalkotob A, Hafezalkotob A, Liao H, Herrera F. Interval MULTIMOORA method integrating interval Borda rule and interval best–worst-method-based weighting model: case study on hybrid vehicle engine selection. IEEE transactions on cybernetics. 2019;50(3):1157–1169. doi: 10.1109/TCYB.2018.2889730 30668492

[44] Gupta H. Evaluating service quality of airline industry using hybrid best worst method and VIKOR. Journal of Air Transport Management. 2018;68:35–47. doi: 10.1016/j.jairtraman.2017.06.001

[45] Faizi S, Sałabun W, Nawaz S, ur Rehman A, Watróbski J. Best-Worst method and Hamacher aggregation operations for intuitionistic 2-tuple linguistic sets. Expert Systems with Applications. 2021;181:115088. doi: 10.1016/j.eswa.2021.115088

[46] Liao H, Gou X, Xu Z, Zeng XJ, Herrera F. Hesitancy degree-based correlation measures for hesitant fuzzy linguistic term sets and their applications in multiple criteria decision making. Information Sciences. 2020;508:275–292. doi: 10.1016/j.ins.2019.08.068

[47] Herrera F, Herrera-Viedma E, et al. A model of consensus in group decision making under linguistic assessments. Fuzzy sets and Systems. 1996;78(1):73–87. doi: 10.1016/0165-0114(95)00107-7

[48] Herrera F, Herrera-Viedma E. Linguistic decision analysis: steps for solving decision problems under linguistic information. Fuzzy Sets and systems. 2000;115(1):67–82. doi: 10.1016/S0165-0114(99)00024-X

[49] Xu Z. A method based on linguistic aggregation operators for group decision making with linguistic preference relations. Information sciences. 2004;166(1-4):19–30. doi: 10.1016/j.ins.2003.10.006

[50] Xu Z. Group decision making based on multiple types of linguistic preference relations. Information Sciences. 2008;178(2):452–467. doi: 10.1016/j.ins.2007.05.018

[51] Liao H, Xu Z, Zeng XJ. Distance and similarity measures for hesitant fuzzy linguistic term sets and their application in multi-criteria decision making. Information Sciences. 2014;271:125–142. doi: 10.1016/j.ins.2014.02.125

[52] Rezaei J. Best-worst multi-criteria decision-making method: Some properties and a linear model. Omega. 2016;64:126–130. doi: 10.1016/j.omega.2015.12.001

[53] Ge S, Wei C. Hesitant fuzzy language decision making method based on 2-tuple. Operat Res Manag Sci. 2017;26:263–274.

[54] Wang L, Wang Y, Liu X. Prioritized aggregation operators and correlated aggregation operators for hesitant 2-tuple linguistic variables. Symmetry. 2018;10(2):39. doi: 10.3390/sym10020039

